# Some Variations in Appendicits

**Published:** 1910-11

**Authors:** Frank K. Boland

**Affiliations:** Atlanta, Ga.


### SOME VARIATIONS IN APPENDICITIS.

By Frank K. Boland, M. D., Atlanta, Ga.

It is not my purpose in this paper to speak of the varieties of appendicitis, as they are usually understood, but to call attention to a few variations in its symptoms and pathology which have come under my personal observation.

We seldom meet disease in exactly the form laid down in the text-books, but I believe this is true of appendicitis more than of any other of the common diseases, unless it be typhoid fever.

The common symptoms of appendicitis are: 1. Sudden pain in the abdomen, usually referred to the right iliac fossa. 2. Fever, often of a moderate grade. 3. Gastro-intestinal disturbance —nausea, vomiting, and frequently constipation. 4. Muscular rigidity and tenderness in the region of the appendix, with or without tumor.

Most cases usually have all these symptoms, more or less marked, but not infrequently it becomes necessary to make a diagnosis of appendicitis in the presence of not more than two of them. I had a case last winter in which the only symptoms were vomiting and obstinate constipation. Indeed, the picture was one of intestinal obstruction rather than of appendicitis, and even after the patient was on the table, I was undecided whether to make the incision in the median line or at McBurney's point. The latter place was chosen, and a badly gangrenous appendix found, the adhesions from which had partially obstructed the gut. The fact that there was almost total absence of fever, pain, tenderness and muscular rigidity made the case unique. It is true that there was a leucocytosis of 15,000 and a relative increase of the polynuclear cells, but these findings do not make a diagnosis of appendicitis, though if the diagnosis is already established, the blood analysis may tell to what extent the disease has advanced. It was later learned that this case was a sequel of amoebic dysentery.

Fever is not a symptom of the greatest importance, except as taken with other symptoms. There is one point in the temperature taught me in hospital experience, which I have never seen in the literature, and that is the difference in the mouth and rectal temperatures. The difference in these temperatures in a healthy person may be as much as half a degree, but in appendicitis it will be a whole degree, if not more. Not long ago, in a case with but slightly marked symptoms, I allowed this one degree to decide me to operate for appendicitis, and the findings justified the procedure. Of course, when an increased rectal temperature is present in women, the possibility of involvement of the pelvic organs must be considered.

Of all the symptoms of appendicitis, pain is the only one which can be said to be constant. The location of the pain, however, certainly is very variable. We have seen it at times referred to all parts of the abdomen. Howard Kelly reports a case in which severe pain was present in the right testicle two days before any other signs of the disease were manifested. Several cases have been reported recently in which the first pain suggested pleurisy.

It must be borne in mind that the value of McBurney's point in diagnosis is as a point of tenderness and not as a point of pain. And even this spot sometimes fails to do its duty as a guide. The most exquisite tenderness may be just above or just below it. I recall a patient in whom the point of tenderness in his first attack was so nearly over the right kidney as to simulate renal colic. In his second attack, a few months later, the tenderness had moved down to a point about two inches above McBurney's. In the third attack, which followed in three weeks, the tenderness was located directly at McBurney's point. The operation, ten days afterwards, revealed gangrenous appendicitis.

The most remarkable variations in the pathology of appendicitis that have come to my notice have been in regard to the connection of the disease with inguinal hernia. I have had three such cases, in none of which was a positive diagnosis made before operation. In one case a man who had been complaining of considerable pain in an old hernia for several days, was operated upon for the radical cure, and an acutely inflamed appendix disclosed adherent to the sac. There had been no other symptoms of appendiceal trouble except the pain, and that was attributed to the irritation of a truss which the patient had been wearing.

The second case was chronic appendicitis in an acutely strangulated hernia, in which it seems failure to recognize involvement of the appendix certainly should be excused. One foot of very dark intestine was in the sac, and the patient was in bad condition when operated upon, though made a good recovery. The appendix was enormously thickened and evidently had been diseased for a long time. It was adherent by a kink to the hernial sac.

The third case was most extraordinary. The patient had a hard, tender lump over the right internal ring, which appeared to be an inflamed gland, a knuckle or gut or an omental hernia. A knuckle of gut seemed most probable since the patient was suffering from partial obstruction. He had never had a hernia before, and the present condition had existed about forty-eight hours. Imagine our surprise when we operated on this man and discovered that this tender mass was a gangrenous appendix, which had slipped through the internal ring. There was no hernial sac, though the bulging appendix had, of course, weakened the internal ring. The tip of the organ lay close to the conjoined tendon, and there was no strangulation by the ring. Another unusual feature was that the appendix had a double mesentery, which should have discouraged inflammation, since this furnished the organ with a double blood supply.

In the dissecting room of the Atlanta School of Medicine, a few years ago, I ran across a variation in the appendix which probably would have puzzled some doctor in diagnosis had it ever been diseased. The cecum and appendix were on the left side, and every viscus of the body was similarly transposed to the wrong side, the sigmoid flexure being on the right, the liver on the left, spleen on the right, apex of the heart on the right, and innominate artery on the left. Had this subject ever had appendicitis, a clue to the condition would have been the discovery of the apex of the heart on the right side, and mapping out the liver dullness on the left side.

We should not forget that appendicitis is the most common of all acute surgical diseases of the abdomen, and that while in the majority of cases its diagnosis is an easy matter, sometimes it appears in a garb so strange that even the most alert examination will not always reveal its presence.

*Discussions on the paper of Dr. F. K. Boland, Atlanta, Ga.—*

Dr. H. B. Disharoon, Roanoke, Ala.—Dr. Boland has completely covered the ground. I recall a case several years ago in which the pain was in the region of the right kidney This patient never complained of any pain over the region of McBurney's point, always complained of pain in the side. Found in operating the adhesion above McBurney's point. Often the close relation to ovaritis puzzles many physicians. Think the place to operate is McBurney's point.

Dr. J. D. S. Davis, Birmingham, Ala.—The lesson this valuable paper teaches is that when we are suspicious, we should go in and find what the trouble is, as the doctor tells us he has operated with and without symptoms. Think it necessary when you have trouble in the abdomen to go in and find what is necessary to be done.

Dr. J. L. Campbell, Atlanta, Ga.—The doctor mentions some variations in regard to temperature taken in the mouth and by rectum. I have a patient now in the sanitarium, and last night her temperature by mouth was 96.4. The nurse phoned me, and I suggested taking it by rectum, and when the house surgeon did so, found it to be 98.2 degrees. That is a very frequent symptom, variation in temperature. There are also variations in the location of pain in appendicitis. One patient suffered from pain in knee, preventing bending leg to body; upon examination found fecal appendicitis, which was removed. I saw another case, and misplaced appendix about a year ago, in which the appendix was found enclosed in the Douglass sac. Dr. Boland forgot to mention one point of pain, not so much pain, as tenderness, frequently diagnosis can be made by pressure on what is known as Morris' point. This is always tender in chronic appendicitis. One other point in acute appendicitis, we have frequent pain in the epigastric region.

Dr. J. L. Sappington, Riverview, Ala.—I want to express my thanks for that paper. I have had about thirty-five cases, that when I am by myself and think about them, I now call appendicits. The first one I had, called it "Cherry Stone Colic," later I began to call it appendicitis. I have treated these thirty-five cases, none of them have been operated on; four have died, the others got well; fifteen of whom have had recurrent attacks. I have treated them with common sense medicinal treatment. Have asked some of them to go to surgeons, but they refused to do so, and begged me to cure them, so I treated them, did my best for them, with the above stated results.

Dr. Boland, inclosing.—I have enjoyed the discussion very much, and have nothing in particular to say in reply. I agree with Dr. Davis, in saying that when we find pain in the abdomen, to open and find out what the trouble is, but we cannot always do this with our private patients.

